# The future of carbon neutrality from a scientific research perspective: an interview with Jing-Hai Li

**DOI:** 10.1093/nsr/nwad007

**Published:** 2023-01-09

**Authors:** Li-Hua Chen

**Affiliations:** Li-Hua Chen is a Professor at Wuhan University of Technology

## Abstract

*Since China announced that it will strive to achieve carbon peak by 2030 and carbon neutrality by 2060, it has become the focus of the whole society. The implementation of carbon peaking and carbon neutrality goals requires a range of revolutionary technologies and involves an array of key scientific questions. A great deal of research has focused on the development of new concepts and innovative technologies on carbon science and sustainable development*.

*NSR recently interviewed Professor Jing-Hai Li about the topic of the future of carbon neutrality science and technology from a scientific research perspective. Professor Li is an academician of the Chinese Academy of Sciences (CAS) at the Institute of Process Engineering, CAS. He is a scientist who has been working on exploring complex systems in chemical engineering by multi-scale methodology for more than 30 years. He proposed the concept of Mesoscience, a new interdisciplinary discipline*.

***NSR*:** The implementation of carbon peaking and carbon neutrality goals will undoubtedly usher in a restructuring of the energy sector. It requires a range of revolutionary technologies and involves an array of key scientific questions. Looking ahead, could you please share with us your views about what these major scientific questions are underlying the carbon peaking and neutrality goals that await breakthroughs and solutions?

***Li*:** The precondition of achieving the carbon peaking and carbon neutrality goals is the structural transition from fossil energy to renewable energy, in addition to structural transformation of other industries. Energy transformation and industrial transformation are indispensable parts of the whole process, complementing each other. These transformations, given their complexity, scope, scale and urgency, have presented tough challenges to the scientific community, involving each and every discipline, and have in turn generated tremendous impetus for science and technology progress. We are facing a number of technical bottlenecks in various fields. I believe there are three crucial levels of research in the relevant fields.

In the first level, we need to create new substances and develop energy transformation processes that are efficient, low-carbon and low-cost to meet carbon peaking and carbon neutrality goals. This will require more profound discoveries in Fundamental Sciences in terms of the law of substance transformation, as well as the quantification and formulation of the relation between multiscale material structure and performance.

In the second level, we will expect Engineering Sciences to create new processes and equipment for industrializing emerging techniques and upgrading and transforming the existing ones, which then gives rise to intelligent and green industrial processes with higher energy efficiency, lower carbon emissions and reduced costs.

**Figure:**
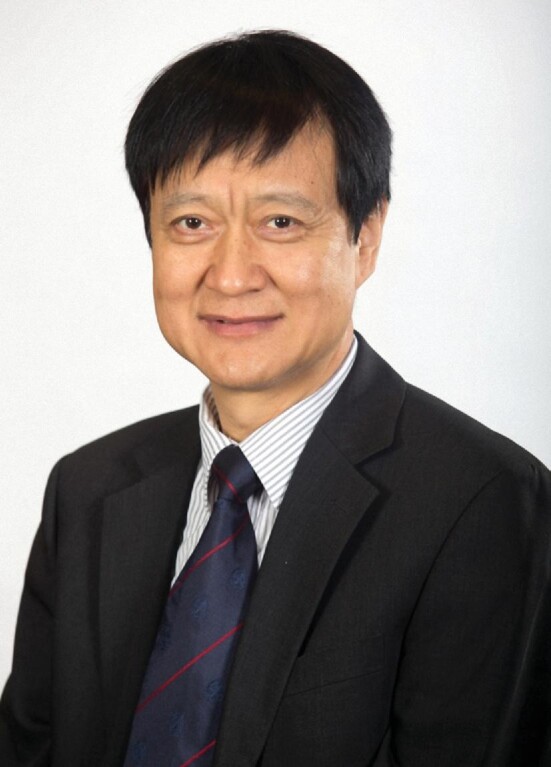
Professor Jing-Hai Li at the Institute of Process Engineering, CAS (*Courtesy of Professor Jing-Hai Li*).

In the third level, we will seek technically feasible system optimization at System Sciences level, optimization of various essential productive factors, consumption patterns, social sectors and industrial structures under the framework of carbon peaking and carbon neutrality goals, so as to pinpoint the optimal pathway to carbon neutrality.

The three levels are mutually bound to each other for system optimization. It is a key issue and should be brought to the scientific community's attention. Despite the striking diversity and complexity in different areas at each level, their common challenge would be structure complexity. From a scientific perspective, the key to achieving carbon peaking and carbon

From a scientific perspective, the key to achieving carbon peaking and carbon neutrality goals lies in the discovery of the common ‘Simplexity’ out of such diversity and complexity of the world.

—Jing-Hai Li

neutrality goals lies in the discovery of the common ‘Simplexity’ out of such diversity and complexity of the world. Therefore, interdisciplinary research is imperative.

***NSR*:** To address the challenging scientific issues of carbon peaking and carbon neutrality goals, Chinese scientists introduced a key notion of green carbon science. What do you think of its role and importance?

***Li*:** I am not so familiar with the concept, but believe that the notion of green carbon science falls into the first level of challenge we just discussed. It reflects leading edge research in Fundamental Sciences under the framework of carbon peaking and carbon neutrality goals, and shows the importance of oxidation-reduction reactions among carbon, hydrogen and oxygen in the transformation of energy resources, carbon reduction and energy efficiency. I believe this notion touches the core of the carbon reduction process. We will need the synergy of relevant disciplines, especially focusing on the quantification and formulation of green carbon science theories, the breakthrough of which would exert a profound influence on carbon peaking and carbon neutrality goals.

To be more specific, it may include ways to enable quantification, regulation and optimization of transformation ratio and selectivity in physiochemical processes, to develop the directional and structural design of relevant materials based on functional requirements and knowledge accumulated through existing experiments, and especially to enhance the overall efficiency in energy transformation, storage and utilization, among others. I personally believe that we’ve primarily met the conditions to solve these problems through interdisciplinary studies that integrate knowledge of various disciplines and focus on scientific advances in formulating dynamic complex structures. Perhaps this is the value of the notion of green carbon science.

***NSR*:** As mentioned earlier, realization of carbon neutrality involves system engineering that involves various aspects. Which aspects do you think need to be specially addressed?

***Li*:** This is a crucial question that requires our full attention. Featuring multiple levels, objectives, stages and disciplines, the realization of carbon neutrality presents itself as a complex system problem that involves natural sciences, engineering, humanities and society, data, management and policy *et al*. The three levels mentioned earlier make up one facet of this system problem, namely science and technology.

As shown in Fig. 1, the complex system model of the implementation of carbon peaking and carbon neutrality goals consists of three levels (regional, national and global), and the most challenging tasks are presented in three parts. To begin with, we need to construct the complex network system shown as part 1, and identify its structural features. It's pivotal to capture the dominant mechanisms of the entire network on the basis of the quantitative relations between each node. Then, we need to establish data information required by part 3 as per requirements by different system levels, and build up its structure and logical system. Last, but not least, we need to define the model expression and content of part 2.

**Figure 1:**
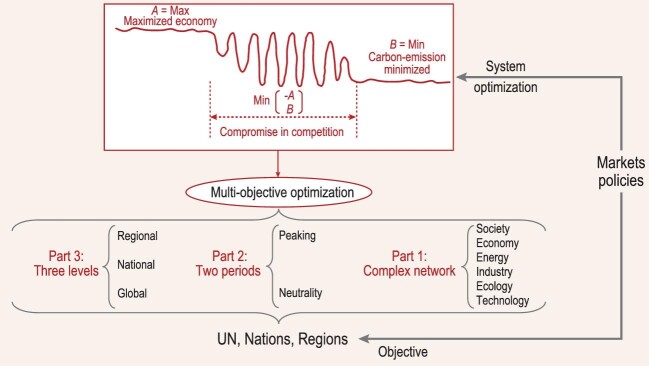
**Figure 1.** A systemic framework for the complex systems of carbon neutrality.

In this way, the implementation of carbon peaking and carbon neutrality goals can be formulated as a multi-objective optimization problem subject to the constraints of the above three parts. To achieve such multi-objective optimization, the key lies in the quantification of the complex network structure of energy, industry, society, ecology and technology, and the formulation of its dominant mechanisms. The solutions may fall into three scenarios. Neither minimum emissions nor maximum economic growth satisfies the requirement of carbon neutrality. Only a reasonable balance between emission and economic growth can

Carbon neutrality is a common challenge faced by humanity. No single nation, region or industry can detach itself from it. We must call for openness, collaboration, inclusiveness and wisdom.

—Jing-Hai Li

lead to sustainable development. As shown at the top of the figure, this is characterized by three regimes and two critical points. The optimal path should be chosen between the two critical points given the specific conditions of the different levels.

Carbon neutrality is a common challenge faced by humanity. No single nation, region or industry can detach itself from it. We must call for openness, collaboration, inclusiveness and wisdom. If we could join hands and establish a common model for the analysis and synthesis of different levels (regional, national and global), it will help countries and even the entire world to determine their routes to achieve carbon neutrality.

***NSR*:** As an advocate of mesoscience, what mesoscale-related scientific problems do you think need to be addressed in carbon neutrality research?

***Li*:** The complexity of nature reveals multilevel features, with each level showing multiscale structures. The complexity of each level is manifested in the mesoscale structure in between the unit scale and the system scale in this level. Many scientific, technological and engineering challenges are attributed to the complexity of mesoscale structures. In research on any structure, defining the stability conditions of that structure satisfies the prerequisite for quantified design, regulation and optimization. This is why we started the complexity research on mesoscale structure in the first place.

Many mesoscale cases we’ve studied have shown that: the complexity of such mesoscale structures is derived from the compromise in competition between different dominant mechanisms, which can be expressed as multi-objective optimization in mathematics. A system with two dominant mechanisms may find solution results in three regimes: the mechanism A-dominated regime, the regime with mechanisms A/B in compromise in competition, and the mechanism B-dominated regime. Regimes dominated exclusively by mechanisms A or B feature a relatively simple structure, while the A/B meso-regime features the most complex structure. It is quite challenging to find an integrated single mechanism directly in this regime. Mechanisms A and B can be identified separately and then synthesized into the stability conditions expressed by a single objective through physical or mathematical principles.

We believe that there is such ‘Simplexity’ existing behind diversity and complexity, so we proposed the idea of mesoscience in hopes that we would be able to verify this common ‘Simplexity’ in more case studies of complex systems.

Apparently, mesoscale structures can be found in many studies on carbon neutrality, which involves various disciplines and levels. If mesoscience is employed to analyze these systems through transdisciplinary studies, we might be able to provide a new angle to solving these challenges, while we can test out ‘Simplexity’, a common attribute of mesoscale structures from diversity and complexity.

Such analysis is crucial in identifying the problem-related level and its corresponding unit and system, and quantifying the dominant mechanisms at the relevant level. We must be mindful of the hierarchical division, as levels should not be misaligned or simply combined. Traditional knowledge, accumulated by a long history, can play a vital role in formulating dominant mechanisms. That is, mesoscience is a possible new angle to view the complexity of the world.

***NSR*:** In the course of scientific research and technological development on carbon neutrality in China, profound changes are taking place in the paradigm of research. Where do you believe these changes are headed?

***Li*:** This question involves crucial global trends in scientific and technological development. It also echoes a characteristic of modern science, which is the interwovenness of significant challenges and paradigm shifts. On the one hand, such key issues as carbon peaking and carbon neutrality goals mount major challenges to traditional theory and methodology, driving the paradigm shifts of science. On the other hand, only paradigm shifts can lead to solutions to those major challenges that existing theories fail to resolve, such as quantifying multilevel complex systems. This is a two-way, interdependent progress, as significant challenges bring about crucial scenarios for application, while paradigm shifts provide new means of breakthrough. This may require the global scientific community to embrace new thinking patterns and pay special attention to development of complexity science, AI, data and computing sciences. We need to enhance open cooperation and interdisciplinary studies, embracing all kinds of new ideas, and encouraging meaningful explorations of all sorts.

